# Long-Term Effect of an Exercise Training Program on Physical Functioning and Quality of Life in Pulmonary Hypertension: A Randomized Controlled Trial

**DOI:** 10.1155/2021/8870615

**Published:** 2021-02-26

**Authors:** Olga Kagioglou, Sophia-Anastasia Mouratoglou, George Giannakoulas, Dorothea Kapoukranidou, Maria Anifanti, Asterios Deligiannis, Aelita Skarbaliene, Arturas Razbadauskas, Evangelia Kouidi

**Affiliations:** ^1^Laboratory of Sports Medicine, Aristotle University of Thessaloniki, Thessaloniki, Greece; ^2^1st Cardiology Department, Aristotle University of Thessaloniki, AHEPA University Hospital, Thessaloniki, Greece; ^3^Department of Physiology, School of Medicine, Aristotle University of Thessaloniki, Thessaloniki, Greece; ^4^Department of Nursing, Faculty of Health Sciences, Klaipeda University Klaipeda, Lithuania

## Abstract

The aim of this study was to evaluate the effects of a 6-month combined aerobic and strength exercise training program on functional and psychological aspects and health-related quality of life in patients with PH and to evaluate its longer-term impact. In total, 22 stable patients (mean age 53.9 ± 13.8, 13 female) with pulmonary hypertension of World Health Organization (WHO) class I-III participated in a nine-month study. They were randomly assigned into two groups: Group A participated in a 6-month combined aerobic and strength exercise training program, whereas Group B remained untrained. All patients underwent physical and psychological assessment at baseline and at month 6 (after completing the exercise program) and physical assessment after 9 months (3 months posttraining). After the 6-month exercise training program, patients of Group A significantly improved their physical (6MWD, STS 10 rep, STS 20 rep, TUG, lower limb strength, cardiopulmonary exercise time, METs, peak VO_2_, VCO_2_, and VE/VCO_2_ slope) and psychological aspects (SF-36, STAI, and BDI). Between the two groups, differences were observed at the 6MWD (95% CI: 36.2-64.6, *η*^2^ = 0.72), STS 10 rep (95% CI: 6.6-2.2, *η*^2^ = 0.4), STS 20 rep (95% CI: 10.8-2.4, *η*^2^ = 0.34), lower limb strength (95% CI: 7.2-3.6, *η*^2^ = 0.38), cardiopulmonary exercise time (95% CI: 0.1-3.3, *η*^2^ = 0.2), and VCO_2_ (95% CI: 0.1-0.5, *η*^2^ = 0.2). Additionally, psychological changes were noted at SF-36, PCS (95% CI: 3.6-14.8, *η*^2^ = 0.35), MCS (95% CI: 1.3-16.1, *η*^2^ = 0.22), TCS (95% CI: 1.3-16.1, *η*^2^ = 0.22), and STAI (95% CI: 1.8-28.2, *η*^2^ = 0.18). The favorable results of exercise were maintained at the 3-month posttraining follow-up assessment. No exercise-induced complications were observed throughout the study. In conclusion, a long-term exercise training program is a safe and effective intervention to improve functional status, psychological aspects, and health-related quality of life in patients with PH.

## 1. Introduction

Precapillary pulmonary hypertension (PH) is a chronic progressive disease in which patients suffer from exertional dyspnea and fatigue, increasing inactivity and physical deconditioning that further exacerbates their symptoms. Despite advances in pharmaceutical therapies, which led to significant improvement in exercise capacity, exertional dyspnea and fatigue remain basic clinical aspects and finally result in difficulty coping with everyday activities. In addition, breathlessness in low effort and sometimes at rest is associated with elevated anxiety levels. Stress hormones such as cortisol, adrenaline, and norepinephrine are the end products of the activation of the hypothalamic-pituitary-adrenal axis and thus are used as physiological measures of stress. Although acute exercise significantly increases the levels of these three major stress hormones, chronic exercise training is found to attenuate the overall response to the stress stimulus [1]. The sensation of dyspnea results to a vicious cycle, in which the avoidance of physical activity leads to exercise capacity limitations with psychological status worsening that further limit patients' health-related quality of life (HRQoL) [2] and engagement to any kind of physical activity [3–5]. This may finally result in physical deconditioning and further increase of dyspnea. Recent evidence has established the improvements of structured exercise programs on cardiopulmonary and peripheral muscle systems and on HRQoL [6]. In 2015, the ESC/ERS Joint Task Force for the Diagnosis and Treatment of PH included supervised exercise training and advice to implement an active lifestyle in the therapeutic guidelines [7]. However, the effects of a long-lasting exercise training intervention on both functional capacity and psychological factors of patients with precapillary PH has yet to be examined. Therefore, the aim of this study was to evaluate the effects of a 6-month combined aerobic and strength outpatient exercise training program on physical functioning, level of anxiety and depression, and HRQoL status in patients with precapillary PH, as well as its impact on physical functioning 3 months after program termination.

## 2. Materials and Methods

### 2.1. Study Population

Inclusion criteria were as follows: stable patients with precapillary PH classified as group 1 (pulmonary arterial hypertension) or group 4 (chronic thromboembolic pulmonary hypertension) inoperable disease, diagnosed according to current guidelines [7]; WHO functional class ≤III; and stable medical and pharmaceutical therapy for at least three months before randomization. Moreover, all patients had to abstain from any form of structured exercise training for at last 3 months before screening. Patients with a history of unstable angina, uncontrolled arterial hypertension, reduced oxygen saturation at rest with ambulatory oxygen therapy requirements, musculoskeletal or neurological impairments, and psychological or cognitive disorders that may affect their participation to the exercise program were excluded from the study.

### 2.2. Study Design

The study was a randomized controlled trial. The sample size was calculated with G∗Power 3.1.9 [8], and after the baseline evaluation, an online statistical computing web programming (https://www.randomizer.org) was used for the randomization process. A random assignment of the 32 participants in blocks of 2 was done. All patients were assessed at baseline and at months 6 and 9. Thus, the study had a 2 × 3 factorial design, with first factor the two groups (intervention (A) or control (B)) and the second factor the three levels of assessment (baseline, after 6 months, and at month 9). The evaluation included clinical history and examination, assessment of anxiety and depression levels, and HRQoL at baseline and after 6 months. After the completion of the 6-month exercise training, patients of Group A were asked to refrain from any structured exercise program to reassess their physical functioning 3 months postintervention. The functional capacity of Group B, who followed their usual lifestyle routine, was also assessed at months 6 and 9.

The study protocol was approved by the Ethics Committee of Aristotle University of Thessaloniki, and the study was conducted in accordance with the Declaration of Helsinki. All patients were informed about the purpose and the procedures of the study and gave written informed consent before randomization.

### 2.3. Process Measurements and Devices

#### 2.3.1. Screening of Anxiety and Depression and HRQoL Assessment

In order to examine the anxiety levels, salivary cortisol was collected before patients get into the measurement procedure. A sample from their saliva was collected and stored in a Salivette kit. Patients were asked to abstain from food, drink, smoke, brushing of teeth, having any form of exercise, and using any corticosteroid or contraceptives for at least two hours before the test. The enzyme-linked immunosorbent assay (ELISA) technique was used for the analysis of the samples [9].

Thereafter, patients were asked to complete the following three questionnaires, which have been translated and validated in Greek language: (a) The state-trait inventory (STAI) is comprised of two subscales with a self-evaluation questionnaire STAI Y1 that describes what patients feel at this moment and a self-evaluation questionnaire Y2 that indicates what patients feel generally. It is used under permission of Mind Garden [10]. Each subscale with a lower score indicates lower levels of anxiety. (b) Beck's depression inventory (BDI) consists of a 21-question, self-reported inventory that measures the severity of depression as the total score rises [11]. (c) The Greek version of the SF-36 is used to assess health-related quality of life. It can be used under permission of Quality Metric Inc. and after accepting the license agreement. The SF-36 is comprised of two scales, all of which have demonstrated high levels of validity, reliability, and stability of scores when administered to patients with PAH. The two summary measurement scales comprise physical health (physical functioning, bodily pain, and role of physical and general health) and mental health (vitality, social functioning, and role of emotional and mental health). Each subscale with a higher score indicates better health [12].

#### 2.3.2. Cardiopulmonary Exercise Testing

Physical functioning assessments were conducted over two consecutive days. On the first day, each patient underwent the maximal cardiopulmonary exercise testing, and on the next day, each one underwent functional capacity assessment. On day 1, patients underwent a symptom-limited cardiopulmonary exercise testing on a Trackmaster Treadmill (Full Vision Inc., Newton, KS) using the Bruce protocol. The electrocardiogram readings and oxygen saturation were continuously recorded throughout the examination, while blood pressure was measured at the end of each stage. Expiration gases were analyzed using MedGraphics Breeze Suite CPX Ultima ergospirometer device (Medical Graphics Corp, MN). Peak oxygen consumption (peak VO_2_) was defined as the highest oxygen consumption obtained, characterized by a plateau of oxygen uptake despite further increases in work rate (steady time). Obtained peak oxygen consumption values were considered maximal when the respiratory exchange ratio was greater than 1, 10. Measurements at peak exercise included systolic and diastolic blood pressure, heart rate, exercise time, oxygen uptake (VO_2_), carbon dioxide consumption (VCO_2_)_,_ anaerobic threshold (AT), pulmonary ventilation (VE), and slope of expired minute ventilation for carbon dioxide output (VE/VCO_2_).

#### 2.3.3. Functional Capacity Assessment

The next day, the six-minute walking test was conducted to evaluate patients' functional capacity and muscle endurance of lower limbs. Patients had to walk for 6 minutes on a marked course (30 m) under continuous pulse oximetry, and the total distance covered at the end of the test was recorded [13]. Exercise intensity was monitored and guided using the Borg Scale Rating of Perceived Exertion. Strength testing (Baseline Leg Dynamometer) was used to assess strength of the lower limbs, and the best out of three efforts made was recorded. Sit-to-stand test was used to quantify functional lower extremity strength, and patients were requested to rise 10 and 20 times consecutively with a rest time between two efforts and the time taken to complete the task was recorded [14]. By timed up and go test (TUG), the time needed for the patient to rise from a chair, walk three meters, turn around, and walk back to the chair was used for the assessment of the patient's functional mobility [15]. To evaluate hand grip strength, a hydraulic hand dynamometer was used and the average of three isometric strength trials for each hand was recorded [16]. All measurements were conducted in the morning, and patients were advised to refrain from smoking, coffee, and alcohol for at least 12 hours before measurements. The researchers who conducted the assessments of participants were blinded to patient group allocation. After the completion of the 6-month period, patients from Group A did not participate at any structured sessions of exercise, to reassess their physical functioning after 3 months.

#### 2.3.4. Exercise Training Protocol

The 6-month structured supervised exercise training program was carried out in the Laboratory of Sports Medicine at Aristotle University of Thessaloniki structured by cardiologists and a physical education teacher experienced in cardiac rehabilitation. The exercise program consisted of three sessions a week, each lasting 45-60 minutes. The intensity was based on the 60%-80% of the heart rate reached at peak oxygen uptake during initial exercise testing. Training intensity was increased gradually with respect to an individual's tolerability and physiological adaptations. Throughout the exercise sessions, heart rate and oxygen saturation were continuously monitored, while blood pressure was measured every 15 min. The exercise routine consisted of a 10-minute warm-up of the upper and lower extremities with stretching and respiratory thoracic expansion exercises to prepare the musculoskeletal system. The main part consisted of aerobic and strengthening exercises and lasted 30-40 minutes. Patients started with a 20-minute interval cycling on a bicycle ergometer or walking on a treadmill. Thereafter, they performed dynamic exercises using a commercial weight machine for shoulder press, bicep curl, triceps extension, and leg flexion-extension in 2 sets of 8-12 repetitions. The last part of each session consisted of 5-10 minutes of stretching exercises for the large muscle-joint group. Patients were also asked to increase their daily physical activity level on the nontraining days. The exercised patients had to attend at least 80% of the exercise training program to be included in the analysis (Table 1).

After the completion of the 6-month exercise training intervention, Group A patients were asked to continue their active lifestyle and to refrain from any kind of supervised structured physical training program. Their daily physical activity was assessed by self-reporting personalized daily physical activity diaries. Patients of Group B were asked to continue their daily routine.

### 2.4. Statistical Analysis

Data were analyzed with the Statistical Package for Social Sciences (SPSS, Chicago, Illinois, USA), version 25.0 software for Windows. QQ plots and the Shapiro-Wilk test were used to examine the normality assumption, while the assumption of the homogeneity of variances was examined with the Levene test. Accordingly, the initial mean differences between the two groups were studied either with the *t*-test or with the Mann–Whitney *U* test, while the chi-square test was used for nominal characteristics. Mean differences within time and between the two groups were analyzed with two-way repeated measures ANOVA, and the effect of each variable that showed statistically significant differences between the two groups at baseline was studied with linear regression. Finally, Pearson's correlation coefficient was used for the study of the association between variables that revealed statistically significant changes over time. Data were expressed as mean ± SD values, and the two-tailed *p* values < 0.05 were considered as statistically significant.

## 3. Results

**Figure 1:**
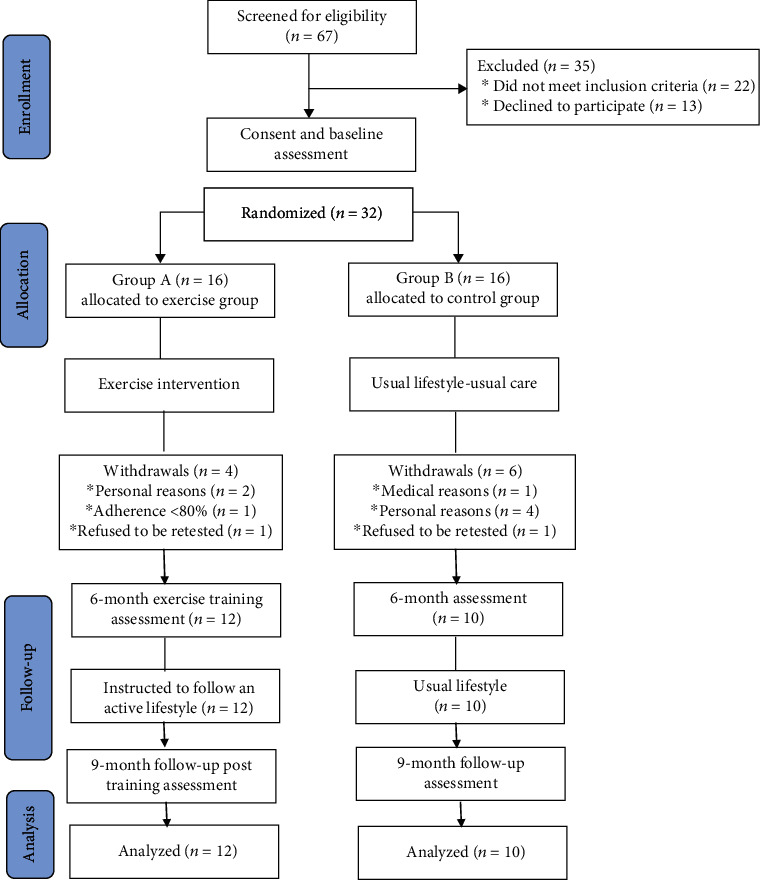
CONSORT diagram of the study design.

A total of 67 patients with documented precapillary PH were recruited from the Cardiology Clinic of AHEPA University Hospital of Thessaloniki, Greece. Of these, 22 patients did not meet the inclusion criteria and 13 declined participation. The remaining 32 patients were randomly assigned into the intervention group (Group A, *n* = 16 patients) and the control group (Group B, *n* = 16 patients). Out of them, 10 patients (4 of Group A and 6 of Group B) withdrew from the study. The flowchart of participants was based on recommendations from the Consolidated Standards of Reporting Trials (CONSORT) and is presented in Figure 1.

Table 2 shows the demographic, clinical, and haemodynamic characteristics as assessed by the right heart catheterization of the patients included in the analysis. At baseline, there were no significant differences between the two groups in respect to anthropometric (sex, age, body mass index, and body surface area), clinical (systolic and diastolic blood pressure, and heart rate), functional characteristics as assessed by 6MWD and cardiopulmonary exercise testing (Tables 3 and 4). Group A patients performed 93 ± 3% of the scheduled sessions, and according to their physical activity diaries, they remained active during the detraining period. No adverse events occurred in any patient during the study period. After the 6-month exercise program, Group A patients showed an increase in the 6MWD by 8.7% (*p* = 0.001), while Group B by 1.6% (*p* = 0.162). In the lower limb strength test, Group A improved by 11.8% (*p* = 0.008), compared to Group B, who did not show any significant change (increased by 0.8%; *p* = 0.881). After 6 months, patients of Group A showed significant improvements by reducing the time in the STS 10-repetition test by 13.7% (*p* = 0.001) and in the STS 20-repetition test by 15.8% (*p* = 0.001), while Group B showed an increase in the STS 10-repetition test by 2.8% and a reduction in the STS 20-repetition test by 2.3%. In the TUG test only Group A showed a significant improvement by 10.1% (*p* = 0.008). Accordingly, after training, Group A showed significant improvements in the exercise time achieved in the cardiopulmonary exercise testing by 51.3% (*p* = 0.002) compared to Group B, who achieved an increase of 6.06% (*p* = 0.048). Additionally, after 6 months, Group A showed a statistically significant increase in METs by 29.4% (*p* = 0.004), peak VO_2_ by 9.5% (*p* = 0.027), and VCO_2_ by 30.7% (*p* = 0.001), while Group B showed an increase only in VCO_2_ by 7.7% (*p* = 0.049). Moreover, the VE/VCO_2_ slope was reduced by 17% (*p* = 0.019) only in Group A.

Additionally, at the end of the 6-month exercise training program, Group A showed a significant decrease in both BDI and STAI scores and improvements in SF-36 results (Table 5).

**Figure 2:**
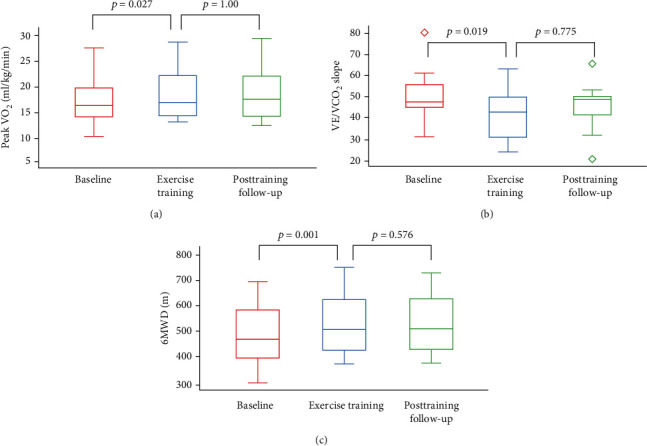
Box plot presenting (a) peak VO_2_, (b) VE/VCO_2_ slope, and (c) 6-minute walk distance of exercised patients at baseline, exercise training period, and posttraining follow-up.

Three months after training cessation, data extrapolated by self-reported dairies showed that patients continued to be physically active. In addition, there was no long-term significant change in their cardiorespiratory efficiency and functional capacity as assessed by the cardiopulmonary exercise test and 6MWD (Figure 2).

Patients allocated in the control group (Group B) failed to demonstrate a favorable improvement in their functional capacity and in their muscle strength (Table 4). Concerning changes between groups, Group A demonstrated a significant increase in the 6MWD, exercise time, and peak VO_2_, as well as on their lower limb strength, after a 6-month assessment, after completion of the exercise protocol compared to Group B patients. Those differences remained significant in the long term, i.e., after a 9-month assessment (Tables 3 and 4).

Finally, Cronbach's alpha showed very good internal consistency for all items, meaning SF-36 (*α* = 0.959), STAI (*α* = 0.892), and BDI (*α* = 0.774).

## 4. Discussion

The results of the present study demonstrate that an outpatient, long-term, combined aerobic and strength exercise training program is feasible and effective in improving physical functioning, overall psychological status, and HRQoL in patients with precapillary PH. Moreover, we went a step further by examining patients' cardiorespiratory efficiency and functional capacity 3 months post intervention, demonstrating that the patients who exercised adopted in the long term to a more active way of life, maintaining the favorable effects of the exercise training program on their functional capacity.

This is the only study to date examining the safety and efficacy of a 6-month mixed-type exercise training program in outpatients with precapillary PH, as the vast majority of the described structured exercise training interventions lasted up to 15 weeks. The only study providing a 10-month exercise training program in PAH patients consisted of only 1.5-hour training once monthly. Although outpatient exercise programs are considered to be less efficient compared to the more intense and closely supervised inpatient exercise protocols, the latter requires the patients' temporal deprivation of their family and everyday activities [17].

We showed that a 6-month exercise training program has exceptional impact on patients' functional capacity. The importance of exercise prescription with regard to its frequency, duration, and type with a combined aerobic, strength, and respiratory muscle training having the most impressive gains is already described and well established [18]. 6MWD is considered as the most frequently utilized tool to assess the effect of structured exercise programs on functional capacity. The majority of the existing studies report diverse results, ranging from lack of improvement to a significant increase in 6MWD [19]. This discrepancy can be explained by the diversity of the described exercise training protocols [20]. Moreover, TUG and STS are reliable tests that have been used in many studies to assess lower limb functional capacity and peripheral muscle performance for patients with different chronic diseases including chronic obstructive pulmonary disease [21]. Interestingly, the strong correlation of these tests with the performance at 6MWD indicates that they can predict not only symptoms and disease severity but also changes in daily life activities and physical performance [22].

The favorable effects of exercise training on respiratory muscle dysfunction, on limited oxygen supply to skeletal muscles, and on quadriceps muscle dysfunction suggest a common pathway for explaining the improvements of exercise capacity [23].

The results of our study demonstrated great improvements after training in both exercise time and peak VO_2_ achieved in the cardiopulmonary exercise testing. Similar results were observed in other studies that demonstrated a significant increase in functional capacity accompanied by a slight increase in peak VO_2_ with the highest increase reaching 15-20% [24]. These changes can be attributed to the beneficial effects of exercise training on the efficacy of muscular gas exchange and metabolism, ventilatory efficiency, and reversal of skeletal muscle atrophy [25] and might include the attenuation of endothelial dysfunction [26] and inflammatory mediators [27] as already documented in chronic heart failure [28]. VE/VCO_2_ slope, which reflects the gas exchange worsening, was significantly reduced in exercised patients, proving the safety and success of the exercise protocol provided [29]. According to recent PH guidelines, the peak VO_2_ and VE/VCO_2_ slope are parameters used in the risk stratification of patients with pulmonary arterial hypertension; thus, their improvement after exercise training may indicate a better overall prognosis in our study population [7].

As exercise intolerance is the most debilitating manifestation of PH, most of the studies focus on its improvement, overlooking its effect in aspects of psychosomatic domains. We demonstrated that the 6-month mixed-type exercise training program led to a significant improvement in anxiety, depression, and overall HRQoL [30]. By improving patients' exercise tolerance and reducing clinical symptoms, such as dyspnoea and fatigue during exertion, exercise training may affect psychological distress and prevent or even improve emotional disturbances and various daily stressors such as depression and anxiety originating from their disease. Improvements in clinical symptoms and physical disabilities can lead to a better emotional state, better social relationships, and an enhancement of HRQoL [31]. In contrast, despite the marked decrease on salivary cortisol levels after our intervention and its increase in the control group after 6 months, we failed to demonstrate a statistically significant association with the levels of anxiety as indicated by the results of the state-trait anxiety inventory (STAI). This is in contrast to the general belief that exercise has beneficial effects on hormone modulation and improved psychological distress [32]. This discrepancy, although unexpected, may be attributed to the intensity of the exercise protocol, as high-intensity exercise regimens are in line with increased levels of cortisol secretion and stress, while moderate exercise protocols seem to have the most positive effects on distress. Another explanation can be the relatively small sample size of the study [33].

Finally, there was no exercise-induced complication throughout the study confirming the safety of the supervised exercise training in patients with precapillary PH. Interestingly, at the 3-month posttraining follow-up assessment, there was no significant deterioration in any cardiorespiratory efficiency and functional capacity parameters. This result indicates that our exercised patients remained active long after the termination of the exercise intervention underlining its beneficial effects not only on patients' physical status but most importantly on embracing a more healthy and active daily life.

The results of our study should be interpreted in light of some limitations. Firstly, the small sample size makes it hard to generalize our findings and to allow further sample stratification according to baseline WHO class. However, precapillary PH is a rare disorder and patients' recruitment for long-term exercise training interventions is a difficult task.

## 5. Conclusions

A long-term combined exercise training program in patients with precapillary PH is safe and feasible with beneficial effects on functional capacity, cardiorespiratory efficiency, anxiety, depression, and HRQoL. A patient's active lifestyle provides an effective way to cope with their functional limitations and should be integrated into the individualized treatment of patient.

## Figures and Tables

**Table 1:** Modified Consensus on Exercise Reporting Template (CERT) for therapeutic exercise interventions.

Item category	Item no.	Abbreviated item description	Therapeutic exercise detail
What: materials	1	Type of exercise equipment	At the exercise program, treadmill and bicycle ergometer, rowing ergometer, and commercial weight machine were used
Who: provider	2	Qualifications, teaching/supervising expertise, and/or training of the exercise instructor	All training sessions were conducted in groups of 6 patients in a fitness gym of the Laboratory of Sports Medicine in Aristotle University of Thessaloniki. Exercise was implemented by a certified cardiac rehabilitation specialist under the supervision of a cardiologist
How: delivery	3	Whether exercises are performed individually or in a group	The rehabilitation session consisted of a group of 6 persons, and the exercised patients received 3 sessions/week, for a total duration of 1 hour
	4	Whether exercises are supervised or unsupervised	All sessions were conducted in an outpatient rehabilitation center, in the fitness gym of the Laboratory of Sports Medicine under the instructions of a cardiac rehabilitation specialist and the supervision of a cardiologist
	5	Measurement and reporting of adherence to exercise	Exercise adherence was recorded by the exercise trainer at every supervised session and was calculated by dividing the number of rehabilitation sessions attended by the number of rehabilitation sessions scheduled
	6	Details of motivation strategies	The motivational program consisted of extensive counseling and information to ensure that patients received clear instructions, emphasizing the importance of regular and consistent exercise. Reinforcement techniques were used, with the physical training instructor giving positive feedback and commending patients for their efforts
	7	Decision rules for progressing the exercise program	The exercise routine consisted of 10 minutes warm-up of upper and lower extremities with stretching and respiratory thoracic expansion exercises to prepare the musculoskeletal system. The first goal was a light to moderate aerobic training of 20-minute interval cycling on a bicycle ergometer or walking on a treadmill that corresponded to 60%-80% of the heart rate reached at peak oxygen uptake, as the strength training performed in a workload of 20-60% of 1 RM for 2-3 sets of 8-12 repetitions of a given exercise. Training intensity was increased gradually with respect to the individual's tolerability and physiological adaptations. The last part of each session consisted of 5-10 minutes of stretching exercises for the large muscle-joint group
	8	Each exercise is described so that it can be replicated (e.g., illustrations and photographs)	The warm-up took place in a sitting position; the aerobic exercise was performed on a cycle ergometer, treadmill, elliptical ergometer, rowing ergometer, or a combination; and dynamic strength exercises consisted of shoulder press, bicep curl, triceps extension, leg flexion-extension, leg extension, leg press, chest press, and seated row
	9	Content of any home program component	All exercised patients were encouraged to increase their daily physical activity level on the nontraining days and be physically active
	10	Nonexercise components	The intervention did not contain any specific education session, except for instructors' support and advise for the health benefits of exercise to increase the adherence of the patients
Where: location	11	Setting in which exercises are performed	The 9-month intervention period consisted of a 6-month structured exercise protocol performed in a nonhospital environment and a 3-month postexercise training termination, while patients were advised to continue their active lifestyle and to refrain from any kind of supervised structured physical training program. Their daily physical activity was assessed by self-reporting personalized daily physical activity diaries
When, how much: dosage	12	Detailed description of the exercises (e.g., sets, repetitions, duration, and intensity)	Total duration of the exercise training protocol: 24 weeks Frequency of sessions: 3 sessions/week Total duration of session: 45-60 min Warm-up: 10 min Main part: 30-40 min, interval aerobic training for 20 min and 20 min strength training with 2-3 sets of 8-10 repetitions (20-60% of 1 RM). Last part: 5-10 minutes of stretching exercises for the large muscle-joint group
Tailoring: what, how	13	Whether exercises are generic (“one size fits all”) or tailored to the individual	The exercise prescription was individualized and in accordance with the ESC/ERS guidelines for the diagnosis and treatment of pulmonary hypertension and the American College of Sports Medicine (ACSM) recommendations for developing and maintaining cardiorespiratory fitness. The exercise program was tailored to the individual, depending on the pathogenesis and severity of pulmonary hypertension. The intensity of the exercise began at 60–80% of the maximal heart rate and workload of 20-60% of 1 RM for all individuals and was gradually increased depending on the subject's response and adaptation to the exercise
	14	Decision rule that determines the starting level for exercise	The exercise protocol was structured in accordance to ESC/ERS guidelines for the diagnosis and treatment of pulmonary hypertension: The Joint Task Force for the Diagnosis and Treatment of Pulmonary Hypertension of the European Society of Cardiology (ESC) and the European Respiratory Society (ERS). Endorsed by: Association for European Pediatric and Congenital Cardiology (AEPC), International Society for Heart and Lung Transplantation (ISHLT), and the American College of Sports Medicine (ACSM) recommendations for heart failure rehabilitation
How well: planned, actual	15	Whether the exercise intervention is delivered and performed as planned	Before each training session, the cardiac rehabilitation specialist described the planned exercise intervention to reach the desirable goal. The exercise intervention was delivered and performed as planned

**Table 2:** Demographic and clinical characteristics of the study population at the beginning, at the end of the 6-month study, and after the 3-month follow-up.

	Group A (*n* = 12)			Group B (*n* = 10)		
	Baseline	6-month follow-up (end of exercise training)	Change between baseline and 6-month follow-up	Baseline	6-month follow-up	Change between baseline and 6-month follow-up
	Mean (SD)	Mean (SD)	Mean (SD)	Mean (SD)	Mean (SD)	Mean (SD)
Gender (F/M)	6/6	—	—	7/3	—	—
Age (years)	54.7 (15.6)	—	—	53.1 (12,1)	—	—
RAP (mmHg)	7.1 (1.9)	—	—	8.2 (1.8)	—	—
mPAP (mmHg)	42.3 (15.5)	—	—	45.1 (8.5)	—	—
BSA (m^2^)	1.9 (0.2)	1.9 (0.2)	1.9 (0.2)	1.9 (0.3)	1.9 (0.3)	1.8 (0.3)
PH classification						
Group 1	8			9		
Group 4	4			1		
WHO FC						
I	0			0		
II	10			10		
III	2			0		
SBP (mmHg)	113 (17)	118 (11)	115 (11)	113 (14)	114 (11)	113 (8.7)
DBP (mmHg)	66.7 (8.6)	96.6 (10.5)	67.9 (12.1)	72 (16.7)	76.5 (13.1)	73.4 (15.2)
HR rest (bpm)	77.8 (9.9)	78.9 (7.8)	74.2 (4.9)	77.3 (7)	75.3 (9.1)	76.6 (6.2)

Data are expressed as mean (SD). BSA: body surface area; DBP: diastolic blood pressure; HR: heart rate; mPAP: mean pulmonary arterial pressure; RAP: right arterial pressure; SBP: systolic blood pressure; WHO-FC: World Health Organization functional class.

**Table 3:** Assessment of functional capacity at the beginning, at the end of the 6-month study, and after the 3-month follow-up.

	Group A (*n* = 12)					Group B (*n* = 10)				
	Baseline	6-month follow-up (end of exercise training)	9-month follow-up (3 months posttraining)	Change between baseline and 6-month follow-up	Change between 6- and 9-month follow-up	Baseline	6-month follow-up	9-month follow-up	Change between baseline and 6-month follow-up	Change between 6- and 9-month follow-up
	Mean (SD)	Mean (SD)	Mean (SD)	Mean (SD)	Mean (SD)	Mean (SD)	Mean (SD)	Mean (SD)	Mean (SD)	Mean (SD)
6MWD (m)	483 (125)	525 (112)^∗^	524 (118)	42.8 (20.1)^a^	-1.8 (6.3)	442 (64)	435 (64)	441 (51.7)	-7.6 (10.6)	4.3 (16.6)
STS 10 rep (s)	26.9 (7.5)	23.2 (6.4)^∗^	24 (5.5)	-3.3 (3.7)^a^	0.8 (1.4)	28.3 (5.3)	29.1 (5.5)	29 (5.3)	0.8 (1)	-0.2 (0.5)
STS 20 rep (s)	49.9 (10.6)	42 (11.1)^∗^	43.1 (10.1)	-7.8 (4.8)^a^	1.1 (1.8)	52 (7.7)	50.8 (8.1)	51.1 (7.5)	-1.2 (4.80)	0.3 (0.8)
TUG (s)	6.9 (1.8)	6.2 (1.3)^∗^	6.6 (1.3)	-0.7 (1.2)	0.4 (0.8)	7.6 (1.2)	7.5 (1.1)	7.4 (1.2)	-0.1 (0.1)	0.1 (0.30)
Hand grip strength R (kg)	24.2 (8.4)	23.7 (6.8)	23.8 (6.8)	-0.2 (1.9)	-0.2 (0.6)	21.6 (5.1)	21.3 (4.5)	20.9 (4.2)	-0.3 (1.8)	-0.3 (0.7)
Hand grip strength L (kg)	22.1 (57.2)	20.9 (6.1)	20.9 (6.1)	-0.8 (3.2)	-0.4 (1.2)	20.1 (27.4)	20.4 (5)	19.8 (3.8)	0.3 (0.6)	-0.6 (1.3)
Lower limb strength (kg)	59.9 (13.6)	67 (13.4)^∗^	66.2 (13.3)	7.1 (11.1)^a^	-0.8 (2.9)	51.2 (9.1)	51.6 (7.7)	50.1 (6.5)	0.4 (2.1)	-1.5 (3.8)

Data are expressed as mean (SD); ^∗^*p* < 0.05: baseline vs. 6-month follow-up; ^a^*p* < 0.05: Group A vs. B. STS: sit-to-stand test; TUG: timed up and go test; 6MWD: 6-minute walk distance.

**Table 4:** Results of cardiopulmonary exercise testing at the beginning, at the end of the 6-month study, and after the 3-month follow-up.

	Group A (*n* = 12)					Group B (*n* = 10)				
	Baseline	6-month follow-up (end of exercise training)	9-month follow-up (3 months posttraining)	Change between baseline and 6-month follow-up	Change between 6- and 9-month follow-up	Baseline	6-month follow-up	9-month follow-up	Change between baseline and 6-month follow-up	Change between 6- and 9-month follow-up
	Mean (SD)	Mean (SD)	Mean (SD)	Mean (SD)	Mean (SD)	Mean (SD)	Mean (SD)	Mean (SD)	Mean (SD)	Mean (SD)
SBP_max_ (mmHg)	153 (21)	150.8 (27.8)	148.3 (18.9)	-1.7 (25.2)	-2.5 (25.0)	153 (19.4)	155 (22.6)	154.4 (22.1)	2.0 (23.1)	-0.6 (1.3)
DBP_max_ (mmHg)	70 (10.4)	65.8 (6.7)	72.9 (9.2)	-4.2 (13.8)	7.1 (13.6)	73 (11.6)	71 (9.9)	70 (8.2)	-2.0 (14.0)	-1.0 (3.2)
HR_max_ (bpm)	157 (30.0)	153.3 (19.7)	159.7 (25.6)	-4.0 (28.3)	6.4 (22.0)	161 (33)	159.5 (25)	160.5 (23)	-1.4 (23.5)	1.0 (3.1)
Exercise time (min)	3.7 (2.4)	5.6 (2.8)^∗^	5.5 (2.0)	1.9 (1.6)^a^	-0.1 (1.6)	3.3 (1.0)	3.5 (1.5)	3.4 (1.5)	0.2 (2.1)	-0.1 (0.1)
METs_max_	6.8 (2.7)	8.8 (3.5)^∗^	8.4 (2.0)	2.0 (2.1)	-0.4 (2.0)	6.3 (1.2)	6.6 (1.7)	6.8 (1.6)	0.3 (2.3)	0.2 (0.4)
Peak VO_2_ (ml/kg/min)	16.8 (5.1)	18.4 (5.6)^∗^	18.3 (5.3)	1.7 (2.6)	-0.1 (1.3)	14.2 (2.1)	14.5 (2.6)	14.1 (2.2)	0.3 (2.2)	-0.3 (0.7)
Peak VO_2_ at AT (ml/kg/min)	12.5 (3.8)	12.5 (4.4)	12.7 (5.2)	0.1 (3.03)	-0.1 (3.7)	10.6 (1.9)	10.9 (1.2)	10.7 (1.1)	0.3 (9.2)	-0.2 (0.4)
VE (l/min)	64.5 (17.7)	68.2 (13.7)	69.8 (15)	3.7 (14.8)	1.6 (5.6)	59.2 (12.2)	61.3 (7.9)	60.8 (7.3)	2.1 (8.9)	-0.005 (0.8)
VCO_2 max_	1.3 (0.4)	1.7 (0.6)^∗^	1.6 (0.5)	0.4 (0.4)^a^	-0.1 (0.3)	1.3 (0.4)	1.4 (0.5)	1.4 (0.4)	0.1 (0.3)	0.02 (0.8)
VE/VCO_2_ slope	50 (11.9)	41.5 (11.1)^∗^	45.4 (11.1)	-8.9 (14.9)	3.9 (11.4)	51 (17)	50.6 (18.2)	50.1 (24.1)	-0.4 (7.3)	-0.5 (34.8)

Data are expressed as mean (SD); ^∗^*p* < 0.05: baseline vs. 6-month posttraining follow-up; ^a^*p* < 0.05: Group A vs. B. DBP_max_: maximum diastolic blood pressure; METs: workload; SBP_max_: maximum systolic blood pressure; VE: ventilation; VE/VCO_2_: slope of expired minute ventilation for carbon dioxide; VCO_2_: volume of carbon dioxide after exhalation per minute; VO_2_ peak AT: peak oxygen consumption at AT; VO_2_ peak: peak oxygen consumption.

**Table 5:** Results of HRQoL, state-trait anxiety inventory, Beck's depression inventory, and salivary cortisol level at the beginning and the end of the 6-month study.

	Group A (*n* = 12)			Group B (*n* = 10)		
	Baseline	6-month follow-up (end of exercise training)	Change between baseline and 6-month follow-up	Baseline	6-month follow-up	Change between baseline and 6-month follow-up
	Mean (SD)	Mean (SD)	Mean (SD)	Mean (SD)	Mean (SD)	Mean (SD)
SF-36 physical health	59.3 (10.0)	69.0 (9.3)^∗^	9.7 (7.3)^a^	59.8 (11.7)	60.3 (9.8)	0.5 (5.3)
SF-36 mental health	50.4 (8.3)	56.09 (11.7)^∗^	5.6 (11.3)^a^	50.5 (9.8)	47.4 (10.0)	-3.1 (3.1)
SF-36 total score	109.8 (17.9)	122.2 (23.0)^∗^	12.4 (19.8)^a^	110.3 (20.4)	107.7 (18.4)	-2.6 (7.2)
STAI form Y1	43.7 (11.2)	36.7 (9.38)^∗^	-3.0 (3.4)	46.6 (10.7)	43.7 (11.2)	-1.3 (8.8)
STAI form Y2	43.8 (5.8)	40.8 (5.9)^∗^	-9.9 (10.4)^a^	47.2 (5.4)	43.8 (5.8)	-0.3 (2.3)
STAI total score	87.4 (15.8)	77.5 (12.5)^∗^	-6.9 (9.0)	93.8 (15.6)	87.4 (15.8)	-1.6 (8.9)
BDI	11.4 (6.2)	8.5 (6.7)^∗^	-2.9 (3.4)	11.7 (5.6)	10.6 (4.2)	-1.1 (4.0)
Salivary cortisol (pg/ml)	28.9 (3.6)	26.6 (3.4)	-2.3 (3.9)	27.2 (5.8)	29.0 (7.9)	1.8 (10.3)

Data are expressed as mean (SD); ^∗^*p* < 0.05: baseline vs. 6-month follow-up; ^a^*p* < 0.05: Group A vs. B. BDI: Beck's depression inventory; SF-36: health-related quality of life; STAI: state-trait anxiety inventory.

## Data Availability

The data used to support the findings of this study have not been made available by the Ethics Committee of Aristotle University of Thessaloniki in order to protect the privacy and anonymity of patients who agreed to participate in this study.
